# Culture of skeletal myoblasts from human donors aged over 40 years: dynamics of cell growth and expression of differentiation markers

**DOI:** 10.1186/1479-5876-3-21

**Published:** 2005-05-12

**Authors:** Andreina Baj, Alessia A Bettaccini, Rosario Casalone, Andrea Sala, Paolo Cherubino, Antonio Q Toniolo

**Affiliations:** 1 Department of Clinical and Biological Sciences, University of Insubria Medical School, 21100 Varese, Italy; 2 Department of Surgery, University of Insubria Medical School, 21100 Varese, Italy; 3 Department of Orthopedics, University of Insubria Medical School, 21100 Varese, Italy

**Keywords:** Cellular therapy, Stem cells, Heart failure, Karyotype, Human viruses

## Abstract

**Background:**

Local myogenesis, neoangiogenesis and homing of progenitor cells from the bone marrow appear to contribute to repair of the infarcted myocardium. Implantation into heart tissues of autologous skeletal myoblasts has been associated with improved contractile function in animal models and in humans with acute myocardial ischemia. Since heart infarction is most prevalent in individuals of over 40 years of age, we tested whether culture methods available in our laboratory were adequate to obtain sufficient numbers of differentiated skeletal myoblasts from muscle biopsy specimens obtained from patients aged 41 to 91.

**Methods and results:**

No matter of donor age, differentiated skeletal muscle cells could be produced in vitro in amounts adequate for cellular therapy (≥300 millions). Using desmin as a cytoplasmic marker, about 50% cultured cells were differentiated along myogenic lineages and expressed proteins proper of skeletal muscle (myosin type I and II, actin, actinin, spectrin and dystrophin). Cytogenetic alterations were not detected in cultured muscle cells that had undergone at least 10 population doublings. Molecular methods employed for the screening of persistent viral infections evidenced that HCV failed to replicate in muscle cells cultured from one patient with chronic HCV infection.

**Conclusion:**

The proposed culture methods appear to hold promise for aged patients not only in the field of cardiovascular medicine, but also in the urologic and orthopedic fields.

## Background

The concept of therapeutic implantation of autologous cells capable of replicating and differentiating into specialized progeny has generated great interest. This approach is being intensively investigated especially in cardiovascular medicine for the regeneration of the infarcted heart [1]. Skeletal muscle has an outstanding ability to respond to the requirements of growth, remodeling, physical activity, and injury. Since adult myofibers are terminally differentiated, the regeneration of skeletal muscle is mostly dependent on a population of resident cells termed satellite cells (SC). These cells lie beneath the basal lamina, but above the plasma membrane of myofibers [2]. When physiologically required, SC become activated, proliferate, differentiate and eventually fuse into myotubes that mature into myofibers [2]. The process of skeletal muscle regeneration is influenced by the nature of injury (e.g., traumatic, chemical, ischemic), the composition of the extracellular matrix, the availability of growth factors produced by inflammatory cells, vascular cells, and motor neurons [3]. SC have a smaller nuclear size compared with the adjacent nucleus of the myotube, and an increased amount of nuclear heterochromatin compared with that of the myonucleus [4]. Quiescent SC show little transcriptional activity. Following activation, they appear as a swelling on the myofiber with cytoplasmic processes that extend from one or both poles of the cell. In adult skeletal muscles, SC have been estimated to represent 1% to 12% of cells in adult muscle. Activation of SC during muscle regeneration requires upregulation of *Myf5 *and *MyoD*, transcriptional activators of the myogenic regulatory factor family (MRF). Expression of these factors determines the transition of myogenic cells into myoblasts [3]. Expression of *Pax3*, *Pax7*, *Sox15*, MNF, cyclooxygenases and other regulatory factors is also needed for SC activation [5-7]. Expression of the late MRFs (myogenin and MRF4) precedes the production of muscle-specific proteins (e.g., myosin heavy chain and muscle creatine kinase). Expression of cytoskeletal (desmin) and surface markers (M-cadherin, Syndecan-3 and -4, VCAM-1, CD56, Glycoprotein Leu-19, CD34) as evidenced by immunostaining is often exploited to follow myogenic differentiation [3,8,9].

Over the past decade, experimental evidence has been accumulating that the implant of cultured myoblasts can represent an effective approach for repairing damaged myocardium [1,10-12]. The functional benefit of intramyocardially transplanted autologous or heterologous skeletal myoblasts has been established in different animal models (rat, hamster, sheep) in which heart damage was determined primarily by ischemia [13,14]. In humans, autologous skeletal myoblast implantation has been practiced only in cases of left ventricular dysfunction produced by ischemic damage [15,16]. Recently, the implant of autologous myoblasts has been proposed also in the setting of dilated cardiomyopathy [17], urinary incontinence [18,19], traumatic muscle damage [20], congenital muscle disease [21]. As compared to other attractive cell types, skeletal myoblasts have several clinically appealing properties, including autologous origin, ease of procurement, the ability of sufficient expansion in vitro, and the ability of adequate differentiation upon in vivo implant.

The present study was initiated to determine if currently available cell culture methods were suitable for obtaining sufficient numbers of differentiated cells from muscle biopsies of human donors aged over 40 years. The latter condition was set since, with the exception of traumatic injury, most of the above listed clinical conditions arise in patients older than 50 years. In this context, it should be borne in mind that, for cellular therapy, substantial numbers of implantable cells (i.e., ≥10^8^) are needed within 1–2 months of disease onset. In addition, cells expanded in vitro need to be adequately differentiated along myogenic lineages, free of demonstrable genetic alterations, and free of contaminant macromolecules [22] and microorganisms.

## Methods

### Cell donors and culture of skeletal muscle cells

Except where specified, chemical reagents, tissue culture media, enzymes and human recombinant growth factors were obtained from Sigma-Aldrich (St. Louis, MO). Plasticware was obtained from Falcon (Becton Dickinson, Milan, IT). Certified double-filtered fetal bovine serum (FBS) produced in a TSE-free country was obtained from HyClone (Logan, UT). Five patients of 41 to 91 years of age gave informed consent for muscle biopsy in the course of programmed heart or orthopedic surgery. Muscle biopsies of at least 1 cm in length were obtained from either the abdominal rectus or femoral quadriceps. Specimens were stripped of visible connective and fat tissue and weighted. Each biopsy provided 0.6 to 1.9 g of muscle tissue. The tissue was immersed in a small aliquot of Hepes-buffered Ham's F12 medium supplemented with imipenem, vancomycin and amphotericin B (at the individual concentration of 10 mg/L), minced with sharp scissors to obtain fragments of approximately 1 mm in diameter. Enzymatic digestion was carried out in Ham's F12 medium containing 1.5 mg/ml Pronase E (nonspecific protease from *Streptomyces griseus*) plus 0.03% EDTA at 37°C for 30 min with occasional shaking. After 5 min sedimentation at 1 g, the supernatant was collected and stored at room temperature. Pelleted tissue debris were subjected to 2–3 further cycles of the above digestion process. Collected supernatants were filtered through a 100 μm nylon mesh (Becton Dickinson) and centrifuged at 400 g for 8 min. The pellet was gently resuspended in proliferation medium (PM). Viable cells were counted by the trypan blue exclusion method. PM consisted of a 1:1 mixture of Dulbecco's modified Eagle's medium and F12 medium (DMEM/F12) containing L-glutamine (2 mM), penicillin (50 units/ml), gentamicin (50 mg/L), and supplemented with: 18% heat-inactivated FBS, gamma-irradiated bovine fetuin (50 μg/ml), recombinant Hu EGF (10 ng/ml), recombinant Hu bFGF (1 ng/ml), recombinant Hu insulin (10 μg/ml), dexamethasone (0.4 μg/ml). Differentiation medium (DM) consisted of DMEM/F12 supplemented with antibiotics, 10% FBS and recombinant Hu insulin (10 μg/ml). Cell suspensions obtained from each biopsy were initially plated in two or more T-75 flasks (i.e., ≥150 square cm) depending on the weight of processed tissue. Cultures were incubated at 37°C in a humidified atmosphere containing 5% CO_2_. Thirty-six hr post-plating, adherent cells were gently washed and the growth medium was changed. Cell passages were performed by light treatment with trypsin-EDTA at the appropriate times indicated by microscopic observation (i.e., 70–80% monolayer confluency). The first passage was carried out 6 to 10 days post-plating. To minimize myogenic differentiation at higher myoblast density, subsequent passages were performed every 3–4 days.

### Cell proliferation

Before each passage, cultures were examined with an inverted microscope equipped with a digital camera. Images were acquired with a 10× objective from 8–10 random fields per culture and recorded. Counts of adherent cells were obtained with a computerized image analysis system (Image DB; Amplimedical, Mira, IT) and expressed as number of cells per square millimeter. After trypsinization, numbers of viable cells were counted by the trypan blue exclusion method. The two methods gave concordant results with an agreement of within 12%. Results are expressed as total numbers of viable cells at different times after culture initiation.

### Immunostaining of muscle cell monolayers

Monolayers of cells that had been expanded for 35 to 40 days in PM were trypsinized and plated with PM in two-well chamber slides (Nunc; PBI, Milan, IT). Two-three days later, after gently removing the medium, cell monolayers were fixed with cold 2.5% paraformaldehyde in phosphate-buffered saline (PBS) for 15 min at 4°C, washed with PBS, permeabilized with 0.1% Triton X-100 in PBS for 15 min, washed again with PBS and blocked for 60 min in TRIS-buffered saline (50 mM Tris, 0.15 M NaCl, pH7.6) containing 0.2% BSA, 0.2% FBS, 0.01% Tween 20, 0.01% sodium azide [immunofluorescence assay (IFA) buffer]. A mouse monoclonal antibody (mAb) to the intermediate filament desmin (clone D33) that is expressed during muscle development was obtained from DakoCytomation (Milan, IT). mAbs to skeletal myosin heavy chain type I slow (clone NOQ7.5.4D; specific for slow myosin HC of skeletal and cardiac muscle), skeletal myosin heavy chain type II fast (clone MY-32; specific for fast myosin HC of skeletal but not cardiac or smooth muscle), A-sarcomeric actin (clone C5C; specific for alpha-skeletal and alpha-cardiac actin), A-sarcomeric actinin (clone EA-53; specific for alpha-skeletal and alpha-cardiac actinin localized at the Z band) were obtained from Sigma-Aldrich. mAbs to spectrin (clone RBC2/3D5; specific for erythrocytes and muscle was used as a marker of membrane integrity) and to the mid rod domain of dystrophin (clone Dy4/6D3; specific for the skeletal, cardiac and smooth muscle forms of dystrophin that anchors the cytoskeleton to the plasma membrane) were obtained from Novocastra Laboratories (Newcastle upon Tyne, UK). Cell monolayers were washed with IFA buffer and incubated 60 min with primary antibody at room temperature. After three washings with IFA buffer, slides were incubated with FITC-labeled sheep anti-mouse IgG (diluted 1:800 in blue Evans-containing buffer) for 60 min in the dark. After three further washings, slides were mounted with an anti-fade containing medium (Vector Laboratories, Burlingame, CA) and examined with a fluorescence microscope (BX60; Olympus, Tokyo, Japan) equipped with a digital camera (Nikon, Tokyo, Japan). Cells showing cytoplasmic staining were counted at 200–400× magnification. Results are expressed as mean percentage of positive cells over the number of examined cells (at least 500 cells per slide were counted; experiments were run in duplicate).

### Cytogenetic analysis

When over 3 × 10^8 ^viable cells were obtained by in vitro culture (i.e., over day-35 post-plating), an aliquot of each culture was trypsinized and plated in two different chamber slides. Cells were grown in PM, treated with colchicine for 30 min, subjected to hypotonic treatment, fixed in methanol:acetic acid (3:1), and processed according to standard methods [23]. Fifty metaphases were analyzed by QFQ-banding with an automated cytogenetics system (Genikon; Nikon) following the rules of the International System for Human Cytogenetic Nomenclature [24].

### Screening of important human viruses

At the time of muscle biopsy, blood samples from each patient were tested for HBV, HCV, and HIV markers by conventional serology (DiaSorin, Saluggia, IT) and by molecular tests (Cobas Amplicor; Roche Diagnostics, Monza, IT). When over 3 × 10^8 ^viable cells had been obtained by in vitro expansion, an aliquot of each culture was tested by gene amplification methods for the presence of important human viruses capable of causing persistent infections. DNA was extracted from a frozen 0.5 ml aliquot of PM containing 3 × 10^6 ^cells using a commercial kit (QIAamp DNA blood mini kit; Qiagen, Milan, IT). PCR was used to detect the genome of DNA human viruses (CMV, EBV, HBV, parvovirus B19). Total RNA was extracted from 3 × 10^6 ^cells by the guanidinium thiocyanate method (Life Technologies, Gaithersburg, MD). cDNA was obtained from 2 μg of RNA with Mo-MLV reverse transcriptase in conjunction with random hexamer primers (Clontech, Palo Alto, CA). RT-PCR was used to detect HIV and HCV genomes. Routine Cobas Amplicor methods were used for HBV, HCV and HIV. Published primers were used to detect CMV, EBV, and parvovirus B19 genomes [25]. The sensitivity of the employed methods was ≤100 genomic equivalents/reaction. CMV, EBV, and parvovirus B19 amplicons were analyzed on 2% agarose gel using ethidium bromide staining and were photographed on a transilluminator with the help of a digital camera (Kodak Image Station 440CF; Celbio, Pero, IT). Clinical samples positive for EBV, CMV, HBV, Parvovirus B19, HCV, or HIV-1 were used as positive controls. Amplification of beta-globin (PCR) or GAPDH (RT-PCR) was used to normalize the data.

## Results

### Growth of primary muscle cell cultures

**Figure 1:**
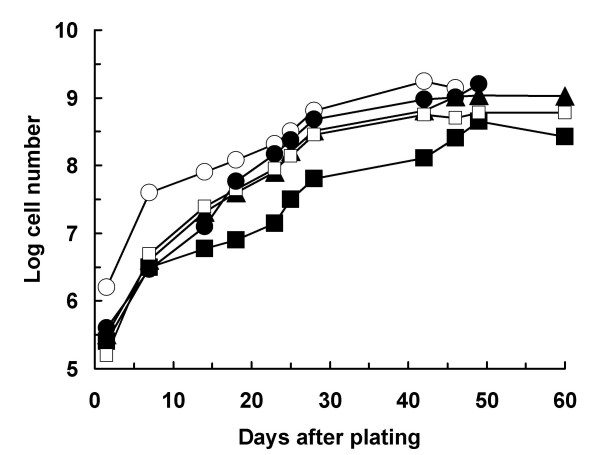
Growth kinetics of primary skeletal muscle cultured in proliferation medium. At different time points, adherent cells were counted as described in the Materials and Methods section. Kinetics of cultures derived from five different patients are reported: (▲) patient #1; (■) patient #2; (□) patient #3; (○) patient #4; (●) patient #5.

As shown in Table 1, the age of muscle cell donors ranged from 41 to 91 years. Tissue obtained from each biopsy weighted from 0.6 to 1.9 g. The kinetics of cell growth using the described culture conditions is illustrated in Figure 1. Thirty-six hr post-plating, the numbers of adherent cells present in primary cultures of the 5 investigated patients ranged from 0.16 to 1.58 millions. Peak cell expansion occurred between day-42 and day-49 of culture. At these times, the total numbers of cultured viable cells ranged from 446 to 1,739 millions. From days 42 to 49 post-plating, the total number of viable cells tended to a plateau. Interestingly, a direct relationship existed between the initial weight of bioptic tissue and the maximal numbers of in vitro expanded cells. This is apparent by comparing tissue weights reported in Table 1 with the kinetic data of Figure 1. The average number of cell population doublings that was required to reach peak cell numbers, was 11.24 ± 0.73 (mean ± SD of 5 different primary cultures). Calculated mean cell doubling times of investigated primary cultures ranged from 3.56 to 4.60 days. Overall, the mean cell doubling time was 3.98 ± 0.38 days (mean ± SD, n = 5). From these data, no relationship was apparent between the age of the cell donor and the mean cell doubling time in culture (Table 1). Under the employed culture conditions, the average fold increase of the number of adherent cells counted on day-1.5 post-plating was 2,490 (range 1,096- to 3,631-fold).

**Table 1:** Patients, weight of muscle biopsy, growth parameters of primary cultures.

**Patient # (gender)**	**Age (yr)**	**Muscle biopsy site**	**Weight of biopsy specimen (g)**	**Mean cell population doubling time (days)**	**Peak number of viable cells (×10^-6^) obtained at the indicated time of culture (day)**
1 (F)	91	Femoral quadriceps	1.0	3.88	1,148 (46)
2 (M)	61	Abdominal rectus	0.6	4.60	446 (49)
3 (M)	41	Abdominal rectus	1.3	3.56	562 (42)
4 (F)^1^	56	Abdominal rectus	1.9	4.15	1,739 (42)
5 (M)	75	Abdominal rectus	1.8	3.74	1,585 (49)

1. Patient showing HCV viremia at the time of muscle biopsy (53,000 genome equivalents/ml).

**Figure 2:**
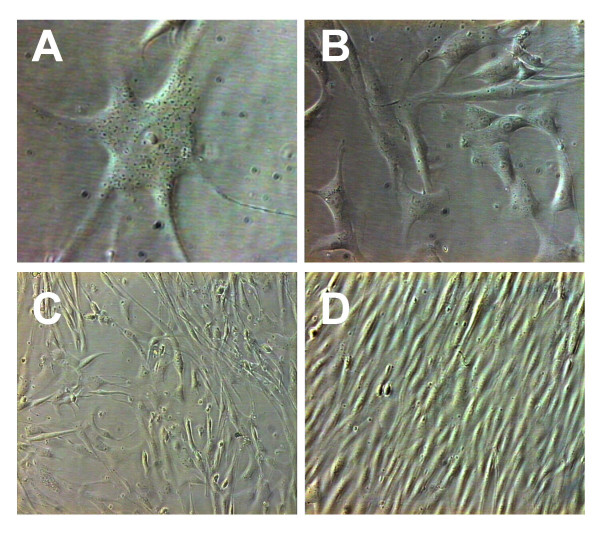
Morphologic aspects of primary human skeletal muscle cultured in vitro. Adherent satellite cell 5 days post-plating in proliferation medium (A; 40×). Semiconfluent monolayers of myoblasts cultured in proliferation medium for 7 (B; 20×) and 10 days (C; 10×). Confluent myoblasts cultured in differentiation medium are in the process of fusing to form myotubes (D; 10×).

Morphologic aspects of primary human skeletal muscle cultures are shown in Figure 2. The morphology of an adherent SC is shown in Figure 2A (day-5 post-plating). Figures 2B and 2C show cell monolayers cultured in PM for 7 and 10 days, respectively. Experience with muscle cultures from adult donors shows that changing the environmental conditions of cultured myoblasts from PM to DM is followed by cell fusion within a few days. Fusion was only used to confirm the presence of myoblasts. Confluent myoblasts in the process of fusing to form myotubes are shown in Figure 2D.

### Expression of differentiation markers by cultured muscle cells

**Figure 3:**
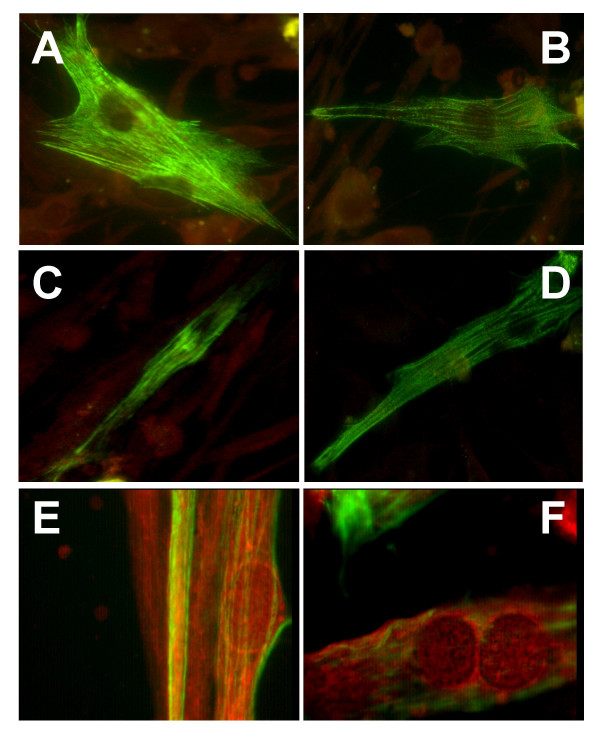
Indirect immunofluorescence to detect the expression of specific markers in primary muscle cells cultured for 35 to 40 days in proliferation medium. Staining of core myofibrillar proteins [myosin type I slow (A) and alpha-sarcomeric actin (B)] produced banded filament images. Staining of myosin type II fast (C) and alpha-sarcomeric actinin (D) produced a continuous filament pattern. The staining pattern produced by spectrin (E) and dystrophin (F) was limited to the periphery of cells and to membrane patches. FITC labeling with Evans blue counterstaining. Original magnification, 60×.

The expression of selected skeletal muscle markers was investigated by indirect immunofluorescence in fixed monolayers of primary cultures expanded in vitro for 35 to 40 days. Table 2 shows the average reactivity of primary cultures with different anti-muscle antibodies. The overall positivity with the anti-desmin mAb (an intermediate filament marker that stains muscle cells, but not fibroblastoid cells) was 49.6% (range 40 to 53%), indicating that approximately half of the in vitro-expanded cell population was differentiated along the myogenic lineage. As shown in Table 2 and Figure 3, antibodies to the core myofibrillar proteins myosin type I slow and A-sarcomeric actin produced banded filament images and stained approximately one-third of cultured myoblasts. Myosin type II fast (specific for skeletal but not cardiac muscle cells) and A-sarcomeric actinin antibodies stained filaments producing a more continuous pattern in 20–30% of cells. The staining pattern produced by spectrin and dystrophin antibodies was remarkably different since was essentially limited to the periphery of cells and to membrane patches in 30–40% of cells.

**Table 2:** Cytoplasmic markers expressed by primary cultures of skeletal muscle cells grown in vitro for 35 to 40 days^1^.

Monoclonal antibody	**Percentage of positive cells (mean ± SD)**^2^	**Fluorescence pattern**
Desmin (skeletal muscle)	49.6 + 6.4	Cytoplasmic
Skeletal myosin type I slow heavy chain (skeletal and cardiac muscle)	33.4 + 3.9	Cytoplasmic filaments
A-sarcomeric actin (skeletal and cardiac muscle)	28.6 + 6.1	Cytoplasmic filaments
Skeletal myosin type II fast heavy chain (skeletal muscle, not cardiac)	29.4 + 6.2	Cytoplasmic filaments
A-sarcomeric actinin (skeletal and cardiac muscle)	19.0 + 3.7	Cytoplasmic filaments
Spectrin (erythrocyte and muscle)	32.6 + 7.5	Peripheral rim and membrane staining
Dystrophin (skeletal, cardiac and smooth muscle)	38.4 + 9.8	Peripheral rim and membrane staining

1. Indirect immunofluorescence assay on monolayers of cells grown using proliferation medium.

2. Positive cells in primary cultures derived from five different tissue donors.

### Karyotype of cultured muscle cells and screening of human viruses

**Figure 4:**
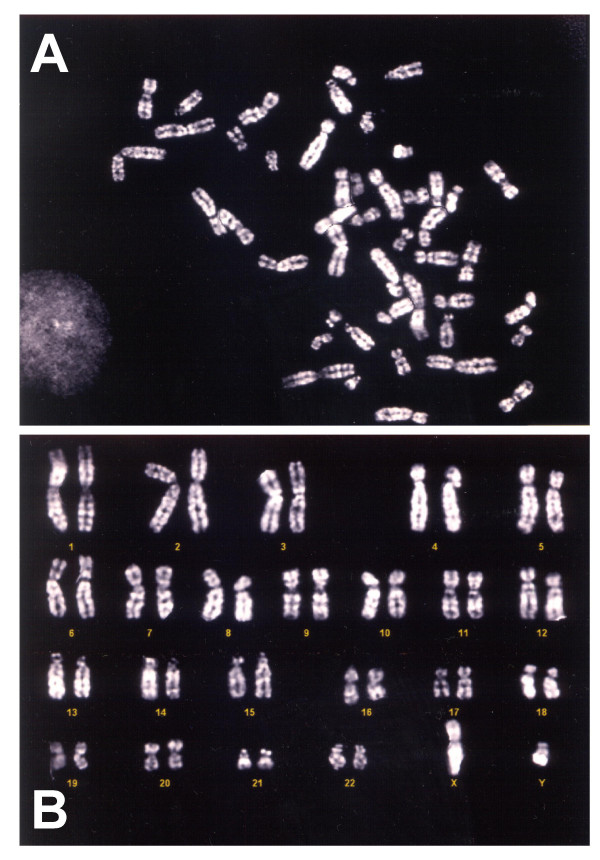
(A) Q banding of a metaphase from the primary muscle cell culture of patient #5 cultured for 35 days in proliferation medium (original magnification, 100×). (B) Normal diploid male cell karyotype of the same patient.

Cytogenetic analysis and the search for human viruses were performed in muscle cell cultures grown in PM for 35 to 40 days. Normal diploid karyotypes were obtained from muscle cultures of all investigated patients. Figure 4 shows a representative metaphase and the normal male karyotype of patient #5.

At the time when cultures were subjected to cytogenetic analysis, samples were also processed for detecting human viral pathogens. After DNA and RNA extraction, PCR and RT-PCR methods were used for detecting the genome of CMV, EBV, HBV, parvovirus B19, HIV, and HCV. All cultures gave negative results with virus-specific primers. Amplification for beta-globin (PCR), GAPDH (RT-PCR), and virus-positive controls consistently gave the expected results (data not shown). At the time of muscle biopsy, RT-PCR had shown that patient #4 was HCV-positive both in blood (53,000 genome equivalents/ml) and in the muscle biopsy specimen. Interestingly, HCV genome was not detected in cultured muscle cells obtained from her biopsy at passage numbers 3, 6, and 9, indicating that HCV failed to replicate in muscle cell cultures.

## Discussion

Currently available tissue culture techniques allow to process skeletal muscle biopsies of patients aged over 40 as to obtain large numbers of isolated (not fused) cells that are differentiated along myogenic lineages. Local myogenesis, neoangiogenesis and homing of progenitor cells from the bone marrow appear to contribute to the repair of acutely infarcted myocardium [16,26]. Implantation of skeletal myoblasts [27,13,28], angiogenetic and protective factors [29-31], hematopoietic stem cells [10] has been associated with improved contractile function in different animal models of myocardial infarction. Implantation of skeletal myoblasts has also been proposed for nonischemic cardiomyopathy [17]. Cautionary notes on the use of bone-marrow derived stem cells for heart regeneration derive from recent experiments in mice in which autologous hematopoietic cells were not capable of differentiating into cardiomyocytes and gave no functional benefits over sham-treated control animals [32,33].

In humans, phase I clinical studies begin to demonstrate the clinical benefits of autologous myoblast transplantation [22,12,34,14,15,11] and, to a minor extent, of autologous mesenchymal/hematopoietic cells [16,35,36].

In order to make clinical applications possible on a larger scale, conditions for the reproducible and safe in vitro expansion of human skeletal muscle need to be set. To this end, five points are of particular relevance: 1) processing of bioptic tissue as to obtain appropriate muscle stem cells; 2) use of culture media free of non-human components; 3) methods to evaluate the differentiation of cultured cells along myogenic lineages; 4) methods to evidence genetic alterations of cells to be implanted; 5) methods to assure the absence of pathogenic microorganisms (viruses causing persistent infections and TSE agents). In the present study, several of these conditions have been satisfied. Bioptic specimens have been processed with a widely available bacterial protease with pleasing results. Preliminary selection of muscle stem cells by fluorescence-activated sorting and/or antibodies has not been attempted in this study, but seems promising [37]. The medium employed has been supplemented with carefully selected human recombinant growth factors. Addition of FBS and fetuin remained however indispensable. Recently, media supplemented with autologous human serum have been proposed and appear to be associated with superior clinical results [22]. In particular, no malignant arrhythmias were reported among 20 patients and the post-infarctual LV ejection fraction was significantly improved. Myogenic differentiation of human myoblasts obtained with the proposed technique has been comparable to what reported by others. Using desmin as a cytoplastic marker, about 50% of cells cultured from adult donors were differentiated along myogenic lineages. This result is comparable to what previously reported using either the desmin marker [9] or the CD56 surface marker [15]. Detailed characterization showed that expanded muscle cell cultures maintained the ability to express proteins proper of skeletal muscle (myosin type I and II, actin, actinin, spectrin and dystrophin). Since the regulation of myosin heavy chain gene expression is strongly regulated by transcriptional events and by physical exercise [38], and since in old subjects muscle fibers co-expressing myosin type I and myosin type IIA are more frequent than in young subjects [39], immunostaining of in vitro cultured cells should not be expected to strictly reproduce what observed in tissue sections.

Of particular interest for clinical applications is that cytogenetic alterations were not detected in cultured cells that had undergone at least 10 population doublings. This was particularly reassuring since the investigated samples were derived from adult/old donors and chromosomal alterations are known to occur at an increased frequency in tissues of adult/old peoples [40]. In future studies, the new CGH technology could offer superior sensitivity for detecting minor cytogenetic changes [41]. Finally, molecular methods for common human viruses are widely available and need to be employed in human clinical trials to validate myoblast cultures prior to implant. Of interest, is the chance observation that HCV failed to replicate in one muscle culture derived from an HCV-infected donor. This result is in agreement with experimental observations showing that HCV has no effects on liver myofibroblasts [42].

## Conclusion

The results indicate that in about one month it is possible to produce in vitro approximately one billion of adequately differentiated skeletal muscle cells from human donors, independently of age. Clinical experience indicates that approximately 300 millions of autologous skeletal muscle cells are sufficient for the cellular therapy of infarcted heart [15,14]. Thus, an early intervention may be possible by processing a muscle biopsy of about 2 grams. The proposed tissue culture methods may also represent a basis on which to envisage applications in the urologic [18,19] and orthopedic fields [20,21].

## List of abbreviations used

BFGF basic fibroblast growth factor

DM differentitation medium

DMEM/F12 Dulbecco's modified Eagle's medium plus Ham's F12 medium

EGF epidermal growth factor

FBS fetal bovine serum

FITC fluoresceine isothiocyanate

HCV hepatitis C virus

IFA immunofluorescence assay

PM proliferation medium

QFQ quinacrine chromosome banding

SC satellite cells

## Competing interests

The author(s) declare that they have no competing interests.

## Authors' contributions

Design and conception of the study (AQT, AS, PC); Development of methods for tissue culture and virus detection (AQT, AB, AAB); Genetic studies of cultured cells (RB); Manuscript preparation (AQT, AB, AS, PC). All authors have read and approved the final manuscript.
